# Whole pelvic helical tomotherapy for locally advanced cervical cancer: technical implementation of IMRT with helical tomothearapy

**DOI:** 10.1186/1748-717X-4-62

**Published:** 2009-12-10

**Authors:** Chen-Hsi Hsieh, Ming-Chow Wei, Hsing-Yi Lee, Sheng-Mou Hsiao, Chien-An Chen, Li-Ying Wang, Yen-Ping Hsieh, Tung-Hu Tsai, Yu-Jen Chen, Pei-Wei Shueng

**Affiliations:** 1 Department of Radiation Oncology, Far Eastern Memorial Hospital, Taipei, Taiwan; 2 Departments of Obstetrics and Gynecology, Far Eastern Memorial Hospital, Taipei, Taiwan; 3 Institute of Traditional Medicine, School of Medicine, National Yang-Ming University, Taipei, Taiwan; 4 Department of Radiation Oncology, Mackay Memorial Hospital, Taipei, Taiwan; 5 Department of Medical Research, Mackay Memorial Hospital, Taipei, Taiwan; 6 Graduate Institute of Sport Coaching Science, Chinese Culture University, Taipei, Taiwan; 7 School and Graduate Institute of Physical Therapy, College of Medicine, National Taiwan University, Taipei, Taiwan; 8 Department of Healthcare Administration, Asia University, Taichung, Taiwan; 9 Department of Education and Research, Taipei City Hospital, Taipei, Taiwan; 10 Department of Radiation Oncology, National Defense Medical Center, Taipei, Taiwan; 11 General Education Center, Oriental Technology Institute, Taipei, Taiwan

## Abstract

**Background:**

To review the experience and to evaluate the treatment plan of using helical tomotherapy (HT) for the treatment of cervical cancer.

**Methods:**

Between November 1st, 2006 and May 31, 2009, 10 cervical cancer patients histologically confirmed were enrolled. All of the patients received definitive concurrent chemoradiation (CCRT) with whole pelvic HT (WPHT) followed by brachytherapy. During WPHT, all patients were treated with cisplatin, 40 mg/m^2 ^intravenously weekly. Toxicity of treatment was scored according to the Common Terminology Criteria for Adverse Events v3.0 (CTCAE v3.0).

**Results:**

The mean survival was 25 months (range, 3 to 27 months). The actuarial overall survival, disease-free survival, locoregional control and distant metastasis-free rates at 2 years were 67%, 77%, 90% and 88%, respectively. The average of uniformity index and conformal index was 1.06 and 1.19, respectively. One grade 3 of acute toxicity for diarrhea, thrombocytopenia and three grade 3 leucopenia were noted during CCRT. Only one grade 3 of subacute toxicity for thrombocytopenia was noted. There were no grade 3 or 4 subacute toxicities of anemia, leucopenia, genitourinary or gastrointestinal effects. Compared with conventional whole pelvic radiation therapy (WPRT), WPHT decreases the mean dose to rectum, bladder and intestines successfully.

**Conclusion:**

HT provides feasible clinical outcomes in locally advanced cervical cancer patients. Long-term follow-up and enroll more locally advanced cervical carcinoma patients by limiting bone marrow radiation dose with WPHT technique is warranted.

## Background

Cervical cancer is the second most frequent cancer among women worldwide [1]. It has demonstrated the superiority of combined chemotherapy with radiotherapy (RT) in the treatment of advanced cervix cancer [2,3]. The radiation therapy consists of external beam irradiation to the primary tumor and corresponding region of lymphatic drainage, followed by brachytherapy to boost the gross tumor in the cervix. A significant benefit of chemoradiation on both overall survival and progress-free survival rate was mentioned [4]. However, grade 3 or 4 haematological (white cell count, 16% *vs*. 8%; platelets, 1·5% *vs*. 0·2%; haematological not otherwise specified, 29% *vs*. 1%) and gastrointestinal toxicities (9% *vs*. 4%) significantly greater in the concomitant chemoradiation group than the RT alone group should also be mentioned. Tan et al. [5] also proposed a late toxicity observation for concomitant chemoradiation of locally advanced cervical cancer. There were 14.5%, 9.4% and 11.4% for grade 3 or 4 urinary, bowel and affecting other organs complications, respectively.

With the advances in radiotherapy modalities, whole pelvic intensity-modulated radiotherapy (WP-IMRT) applied to gynecologic malignancies with excellent planning target volume (PTV) coverage and is associated with less acute gastrointestinal sequelae than conventional whole pelvic radiotherapy (WPRT) as reported by Mundt *et al*. [6]. Under similar target coverage, IMRT is superior to conventional techniques in normal tissue sparing for the treatment of cervical cancer and a number of groups have explored IMRT in the gynecologic setting as a method to minimize the gastrointestinal, genitourinary, and bone marrow toxicity that occurs in conventional RT [7-11].

Helical tomotherapy (HT) is a new CT-based rotational intensity modulated radiotherapy and provides an impressive ability for highly conformal dose distributions and simultaneous critical organ sparing ability [12,13]. HT is being tested to apply for gynecologic malignancies recently and provides encouraging results about excellent setup accuracy and reducing margins for the external beam treatment of gynecologic malignancies [14]. However, this report did not provide the clinical results about the gynecologic malignancies treated by HT.

In our institute, a Tomotherapy Hi-Art system (Tomotherapy, Inc., Madison, Wisconsin, USA) was installed and used for treatment from November 2006. We report here our initial clinical 2 years experience for patients with locally advanced cervical cancer with HT, focusing on the correlation between dosimetry, clinical outcome and early toxicities.

## Methods

### Patient's characteristics

Between November 1st, 2006 to May 31, 2009, 10 patients undergoing whole pelvic HT (WPHT) for locally advanced cervical cancer without pelvic or paraarotic lymphadenopathy at Far Eastern Memorial Hospital (FEMH) were retrospectively enrolled. Staging investigations included complete history and physical examination, fiberoptic endoscopic evaluation, complete blood counts, liver and renal function tests, chest X-ray, magnetic resonance imaging (MRI) scans or computed tomography (CT) scans of the pelvic region. The disease was staged according to the International Federation of Gynecology and Obstetrics (FIGO) criteria [15].

### Radiotherapy

Radiotherapy was administered to the whole pelvic region in 28 fractions totaling 50.4 Gy followed by intracavitary brachytherapy. The total dose of brachytherapy delivered was 30 Gy/6 fractions in patients. The total dose delivered to point A (a reference location 2 cm lateral and 2 cm superior to the cervical os) was 80.4 Gy in patients; the total dose delivered to point p (the pelvic wall) was 55.0 Gy in patients. Cisplatin (CDDP) was administered during external radiation, beginning on the first day of radiation for 5 weeks concurrent with WPHT. A dose of 40 mg/m^2 ^CDDP (maximum dose, 70 mg) was used and administered via a peripheral vein to patients.

### Immobilization

A BlueBAG™ immobilization system (Medical Intelligence, Schwabmünchen, Germany) was used for each of these patients to fix pelvic and extremities. Positioning was supine with arms up, and feet placed in an ankle holder. All patients underwent a CT planning scan with our departmental scanner (Siemens Somatom Plus 4 CT scanner) from the diaphragm to 5 cm below the ischial tuberosities. Localization marks were placed on anterior and lateral sides of the patients at the mid-plane and midline at the level of L4-L5 vertebral body interspace. CT with 5-mm slice thickness was taken for treatment planning. Target objects and normal structures were contoured on a Pinnacle3 treatment planning system (Philips Healthcare, Madison, Wisconsin, USA). The MRI or CT images were retrieved on a Pinnacle workstation and fused with the CT images for contouring of the tumor volume.

### Delineation of target volumes

Delineation and constraints was according to Radiation Therapy Oncology Group (RTOG) 0418 protocol and the International Commission on Radiation Units and Measurements reports 50 [16] and 62 [17] recommendations. The Gross Tumor Volume (GTV) was defined as all known gross disease determined from CT, clinical information, and MRI. The Clinical Target Volume (CTV) was defined as areas considered containing potential microscopic disease. Internal Target Volume (ITV) was defined as the volume of the vagina and paravaginal soft tissues that is in both the empty and full bladder CT scans that were done at the time of simulation and fused together. The Planning Target Volume (PTV) would provide a 7 mm margin (anteriorly, posteriorly, laterally, as well as in the superior and inferior directions) around the nodal CTV and ITV. The treatment plan would be done on the full bladder scan. The treatment plan used for each patient would be based on an analysis of the volumetric dose, including dose volume histogram (DVH) analyses of the PTV and critical normal structures. The GTV plus a 7-mm expansion was defined as the primary tumor CTV to account for microscopic spread, excluding the bowel, bladder, and rectum if they were not clinically involved); The nodal CTV should include the internal (hypogastric and obturator), external, common iliac lymph nodes perinodal tissue, pertinent clips and down to the level of S3. Identification of the CTV usually began with the identification of the iliac vessels. The average margin would be 7 mm. Bone and intraperitoneal small bowel should be excluded from the CTV; also, iliopsoas muscle that lies adjacent to clinically negative lymph nodes should also be excluded from the CTV. Approximately 1.5 cm of tissue anterior to the S1, S2 and S3 sacral segments was usually added to the CTV in order to include the presacral lymph nodes and uterosacral ligaments. The most antero-lateral external iliac lymph nodes that lied just proximal to the inguinal canal should be excluded from the CTV. The CTV of the nodes should end 7 mm from L4/L5 interspace to account for the PTV. The PTV for nodes stopped at L4/L5 interspace. The vaginal and parametrial CTV should actually be an ITV, which will account for internal organ motion. The inferior limit was usually around the level of the upper third of the symphysis pubis but could be individualized based on inferior spread of the patient's tumor. The lateral margin of the vaginal PTV should be to the obturator muscle. However, at least 3 cm of the vagina needed to be treated or at least 1 cm below the obturator foramen. The 90% isodose surface covered between 95% and 98% of the PTV 50.4, or volumes of overdose exceed 115% < 5% of the PTV 50.4 volume could be considered acceptable. The field width, pitch, and modulation factor (MF) usually used for the WPHT treatment planning optimization were 2.5 cm, 0.32 and 3.0, respectively. All patients received daily megavoltage computed tomography (MVCT) acquisitions for setup verification [18].

Normal structures will be contoured using the full-bladder CT scan. The OARs (i.e., bladder, rectum, sigmoid, small bowel, and femoral heads) were contoured as solid organs. Dose-volume constraints for normal tissues were as follows: small bowel (2 cm above the most superior vessel contour) < 30% to receive ≥ 40 Gy, minor deviation 30% to 40 Gy; Rectum < 60% to receive ≥ 30 Gy, minor deviation 35% to 50 Gy; Bladder < 35% to receive ≥ 45 Gy, minor deviation 35% to 50 Gy; Femoral head ≤ 15% to receive ≥ 30 Gy, minor deviation 20% to 30 Gy.

### Intracavitary brachytherapy

An iridium-192 (high-dose-rate) source was used with standard Fletcher-Suit-Delclos intracavitary applicators. Patients were treated twice a week after WPHT completed for 3 weeks, with a prescribed dose of 500 cGy per fraction to Point A. The high-dose rate (HDR) source dwell times were manually calculated based on our institutional system of empiric intracavitary irradiation rules. Postimplantation dosimetry was performed with the GENIE treatment planning system v1.0.4 (Nucletron, Netherland), and included calculation of dose to the "classical" Point A bilaterally (a reference location 2 cm lateral and 2 cm superior to the cervical os), pelvic sidewall bilaterally (Point P, defined as the point 2 cm above the top of the colpostat and 6 cm lateral to midline), and the rectal point and bladder point as defined by the International Commission on Radiation Units and Measurements [19]. For each implant, point doses to Points A and P, the bladder point, and the rectal point were recorded; after completion of therapy, the doses for the six implants were summed. There is no standard or universally accepted fraction size for HDR brachytherapy. At our institution we have chosen to use the fraction size of 500 cGy.

### Conventional treatment planning for comparison

Conventional whole pelvic radiation therapy (WPRT) plans were generated using Pinnacle3 treatment planning system (Philips Healthcare, Madison, Wisconsin, USA). The isocenter was placed at the geometric center of the PTV. A 4-field "box" plan was designed using 6-MV photons with apertures shaped to the PTV in each beam's eye-view. The pelvic field extended from the upper margin of L5 to the midportion of the obturator foramen or the lowest level of disease, with a 2-cm margin, and laterally 1.5 cm beyond the lateral margins of the bony pelvic wall (at least 7 cm from the midline). For the lateral fields, the anterior border was the pubic symphysis and the posterior border was the space between S2 and S3. The fields could be modified to include areas of known tumor and wedges were used as needed. All plans were normalized to cover 98% of the PTV with 50.4 Gy. The 2% underdose represents those voxels at the periphery. This normalization provided conformal coverage while minimizing dose nonuniformity within the target.

### Dose-volume analysis of treatment plans

Dose-volume histograms (DVHs) of the PTVs and the critical normal structures were analyzed accordingly. For PTVs, we evaluated the volume, the volume covered by 95% of the prescription dose (V95), and the minimum doses delivered to 5% (D_5_) and 95% (D_95_) of the PTV. The critical organs with functional subunits organized in a series were examined. The conformal index (CI) and the uniformity index (UI) had been used to evaluate the conformity and uniformity of the plan. The volume received the mean dose for PTV generated from the DVH. The conformal index (CI) for PTV was calculated using the formula CI_ICRU _= *V*_*TV*_/*V*_*PTV*_, where *V*_*TV *_was the ratio of the treated volume enclosed by the prescription isodose surface and *V*_*PTV *_was the planning target volume [17]. The uniformity index (UI) was defined as UI = *D*_5_/*D*_95_, where D_5 _and D_95 _were the minimum doses delivered to 5% and 95% of the PTV reported previously [20].

### Toxicity

Interruptions in radiotherapy might be necessitated by uncontrolled diarrhea, or other acute complications. If radiation therapy was held, then chemotherapy would also be held. Chemotherapy stopped at the completion of RT. If chemotherapy was held, radiation therapy would continue. Radiation was only stopped in cases of grade 4 hematologic or non-hematologic toxicity until toxicity resolved to at least grade 3. CDDP was withheld in any case involving grade 3 toxicity until the toxicity regressed to any grade of <3; in patients with grade 3 toxicity that persisted >2 weeks, chemotherapy was no longer administered.

### Follow-up

Upon treatment completion, patients were evaluated every 3 months for the first year, every 4 months during the second year, every 6 months during the third year, and annually thereafter. At each visit, a physical and pelvic examination, blood counts, clinical chemistry, and chest x-rays were performed. Computed tomography (CT) scan, ultrasound (US), and other imaging studies were conducted when appropriate. Suspected cases of persistent or recurrent disease were confirmed by biopsy whenever possible. Acute and late toxicities (occurring >90 days after beginning RT) were defined and graded according to the Common Terminology Criteria for Adverse Events v3.0 (CTCAE v3.0).

### Statistical methods

Descriptive statistics (mean, median, proportions) were calculated to characterize the patient, disease, and treatment features as well as toxicities after treatment. The overall survival (OS), progression-free survival (PFS), locoregional progression-free (LRPF), and distant metastases-free (DMF) rates were estimated using the Kaplan-Meier product-limit method. Progression was defined as a 50 percent increase in the product of the two largest diameters of the primary tumor or metastasis. Progression-free survival was calculated from the date of pathologic proof to the date of the first physical or radiographic evidence of disease progression, death, or the last follow-up visit. Survival was calculated from the date of pathologic proof to the date of death or the last follow-up visit. All analyses were performed using the Statistical Package for the Social Sciences, version 12.0 (SPSS, Chicago, IL, USA).

## Results

### Patient characteristics

Ten women were included. They had a median age of 58 years (range, 33-72 years). All belong to FIGO Stage IIB and IIIB. The medium tumor volume was 45.9 cm^3^. The medium weekly cycles of chemotherapy were 5 weeks. Seventy percent of patients could complete 4 weekly cycles of chemotherapy. All of the patients were treated with definitively concurrent chemotherapy with WPHT followed by brachytherapy. (Table 1)

**Table 1:** Patient characteristics

Variable	No. of patient (%)
*Age (years)*	
Median (range)	58 (33-72)
*Gender*	
Female	10 (100%)
*Karnofsky performance status*	
< 70	0
≥ 70	10 (100%)
*Pathology*	
Squamous cell carcinoma	7 (70%)
Adenocarcinoma	3 (30%)
*International Federation of Gynecology and Obstetrics (FIGO) stage*	
Stage IIB	9 (90%)
Stage IIIB	1 (10%)
*Tumor size*	
Medium length (range)	5.5 cm (4.3 - 8.4 cm)
Medium depth (range)	3.7 cm (2.4 - 4.6 cm)
Medium width (range)	4.4 cm (3.5 - 6.0 cm)
Weekly cycles of chemotherapy	
5 weeks	5 (50%)
4 weeks	2 (20%)
3 weeks	1 (10%)
2 weeks	2 (20%)

### Treatment outcome

The mean survival was 25 months (range, 3 to 27 months). The actuarial 2-year overall survival, progress-free survival, locoregional control and distant metastasis-free rates were 67%, 77%, 90% and 88%, respectively. The 2-year survival, progression-free, locoregional-progression-free and distant metastasis-free patient number over all patients are 9/10, 8/10, 9/10 and 9/10, respectively. Ninety percent of patients were surviving at the time of this report.

### Dose-volume analysis and comparison for WPHT and WPRT

**Figure 1:**
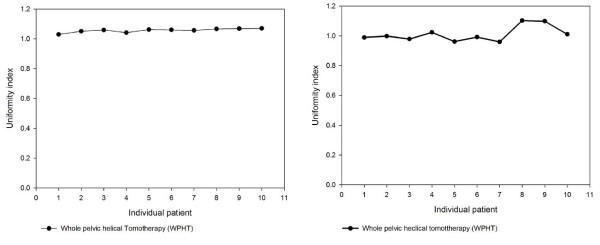
**(A) The uniformity index of helical tomotherapy for 10 patients with locally advanced cervical cancer**. **(B) The conformal index of helical tomotherapy for with locally advanced cervical cancer.**

The WPHT for UI and CI was 1.07 ± 0.05 and 1.01 ± 0.05, respectively. The UI and CI for individual patient are plotted in Figures 1A and 1B, respectively. Dose-volume histograms statistics for the organs at risk are described in table 2. WPHT provided better critical organs sparing than WPRT in the mean dose and the other parameters for rectum, bladder and intestine with a statistically significant level (*p *value < 0.01), respectively. WPHT provided impressive ability of high dose declining for OARs than WPRT. However, WPHT had poorer results for right and left side pelvic bone sparing than WPRT due to lacking of V10 and V20 constraint for planning initially.

**Table 2:** Dose-volume histograms statistics for the organs at risk

		Average ± *S.D.			
		
Organ	Volume (ml) ± *S.D.	Helical tomotherapy	Conventional radiotherapy	†Decreasing percentage	*p *value
*Rectum*	43.5 ± 18.2				
Mean dose		41.3 ± 5.1 Gy	50.9 ± 1.9 Gy	18.9%	< 0.01
V50.4		37.2 ± 30.1%	80.8 ± 12.4%	55.6%	< 0.01
V40		68.3 ± 20.9%	95.2 ± 4.2%	35.0%	< 0.01
V30		82.2 ± 15.3%	98.4 ± 2.6%	16.6%	< 0.01
*Bladder*	59.8 ± 24.2				
Mean dose		40.5 ± 3.5Gy	50.2 ± 2.5Gy	19.3%	< 0.01
V50.4		29.5 ± 14.7%	74.4 ± 17.6%	61.3%	< 0.01
V45		49.1 ± 13.7%	86.0 ± 11.5%	43.2%	< 0.01
V40		57.9 ± 12.6%	91.3 ± 8.5%	36.8%	< 0.01
V30		75.7 ± 12.3%	100.0 ± 0%	24.3%	< 0.01
*Intestine*	1523.3 ± 1389.4				
Mean dose		25.1 ± 2.4Gy	34.2 ± 4.2Gy	26.3%	< 0.01
V50.4		0.4 ± 0.4%	20.0 ± 10.7%	98.2%	< 0.01
V40		4.9 ± 3.2%	33.3 ± 13.1%	84.1%	< 0.01
V30		23.5 ± 11.9%	59.5 ± 10.4%	61.1%	< 0.01
V20		69.2 ± 10.9%	86.6 ± 8.0%	20.1%	< 0.01
*Right femur*	114.4 ± 16.2				
V30		15.5 ± 14.2%	23.2 ± 29.1%	19.0%	0.47
*Left femur*	114.3 ± 14.0				
V30		16.1 ± 13.9%	22.3 ± 28.5%	12.9%	0.54
*Left pelvic bone*	187.3 ± 19.4				
V10		99.9 ± 0.1%	93.1 ± 4.8%	-6.8%	< 0.01
V20		79.1 ± 4.6%	86.2 ± 5.6%	8.2%	< 0.01
*Right pelvic bone*	189.4 ± 20.1				
V10		99.9 ± 0.1%	95.5 ± 2.1%	-4.4%	< 0.01
V20		78.3 ± 4.8%	89.2 ± 3.1%	12.2%	< 0.01

*S.D.: standard deviation.

† Decreasing percentage: (conventional radiotherapy - helical tomotherapy)/conventional radiotherapy

### Acute and subacute toxicity

Acute toxicity of radiation therapy within chemotherapy and late toxicity is detailed in Additional file 1. One grade 3 of acute toxicity for diarrhea, thrombocytopenia and three grade 3 of leucopenia were noted during CCRT. Only one grade 3 of subacute toxicity for thrombocytopenia was noted. There was no grade 3 or 4 subacute toxicities for anemia, leucopenia, genitourinary or gastrointestinal.

## Discussion

In our preliminary results of locally advanced cervical cancer receiving WPHT concurrent with chemotherapy followed by brachytherapy, HT provides feasible outcomes and acceptable toxicity during and after CCRT.

The 2-year estimate of OS, PFS, locoregional failure only and distant metastasis only rate in the RT plus weekly CDDP reported by randomized trials was 67 - 71%, 64 - 84%, 10 - 25% and 6 - 11%, respectively [2,3,21]. The overall survival, disease-free survival, locoregional failure and distant metastasis rate at 2 years in our institute are 67%, 77%, 10% and 12%, respectively. The clinical results of WPHT concurrent with weekly CDDP following by HDR brachytherapy at our institute suggest WPHT is feasible for locally advanced cervical carcinoma patients.

Adding more beams would lead to improved conformality without affecting the value of the objective function [20]. The CI is usually larger than 1, indicating that a portion of the prescription dose was delivered outside the PTV. The greater the CI, the less is the dose conformity to the PTV [20]. The greater UI indicates higher heterogeneity in the PTV [22]. In the current study, the UI and CI for WPHT was 1.07 ± 0.05 and 1.01 ± 0.05, respectively. WPHT provides the impressed conformality and uniformity for locally advanced cervical carcinoma patients. The UI and CI for individual patient are described in Fig. 1A and 1B, respectively.

Despite the clear efficacy of a combined modality approach in locally advanced cervical cancers [2,3,21], toxicity can be considerable. For locally advanced cervical cancer treated with CCRT, the rates of grade 3 acute toxicities for GI effects were 7 - 9% [2,3,23]. For moderate acute hematologic effects, the happening rate during CCRT was reported from 23% to 37% [2,3,23]. In the current study, the moderate acute toxicities during CCRT are listed as follow: one (1/10) for diarrhea, three (3/10) for leukopenia and one (1/10) for thrombocytopenia. (Additional file 1) The acute toxicities of GI and GU for locally advanced cervical cancer treated by WPHT are feasible however the dominant hematologic toxicities are noted in the current study. The late moderate toxicities for locally advanced cervical cancer patients treated with CCRT that reported by previous series are 9.4 - 13% for GI effects and 3 - 14.5% for genitourinary effects [5,21,23]. In the current study, the subacute grade 3 toxicity is only 1 (10%) for thrombocytopenia and there are none with GI and GU effects. (Additional file 1) Compared with WPRT, WPHT decreases the mean dose to rectum, bladder and intestines successfully. In addition, the V50 decreasing percentage for WPHT in rectum, bladder and intestine is 56%, 61% and 98%, respectively. (Table 2) From the view of physics, WPHT decreases the mean and high doses to the OARs entirely when compared with conventional technique and these physic properties of WPHT reflect the declining rate of acute and subacute toxicities for gastrointestinal and genitourinary events successfully. (Additional file 1)

There are numbers of groups that explored how IMRT can minimize the gastrointestinal, genitourinary and bone marrow toxicity than conventional RT for gynecologic cancer patients. When using IMRT techniques for gynecologic treatment, V40 and V30 for the intestine, bladder and rectum is 25 - 40% and 40 - 57%, 65 - 86% and 88 - 97%, 74 - 84% and 87 - 95%, respectively [24-27]. (Additional file 2) Compared with previous reports, HT decreases 80 - 88% of V40 and 40 - 60% of V30 for the intestine, 11 - 33% of V40 and 14 - 22% of V30 for the bladder and 8 - 19% of V40 and 6 - 14% of V30 for the rectum than previous IMRT reports, respectively. It also notes that HT decreases 35% of V45 for the intestine than previous IMRT reports simultaneously. In other words, HT provides significantly superiority for decreasing high dose to these OARs than IMRT does. Therefore, we suggest when treating the locally advanced cervical cancer patients with HT, the optimization constraints of V40 and V30 for the intestine, bladder and rectum could be reconsidered as 5% and 24%, 58% and 76%, 68% and 82%, respectively.

**Figure 2:**
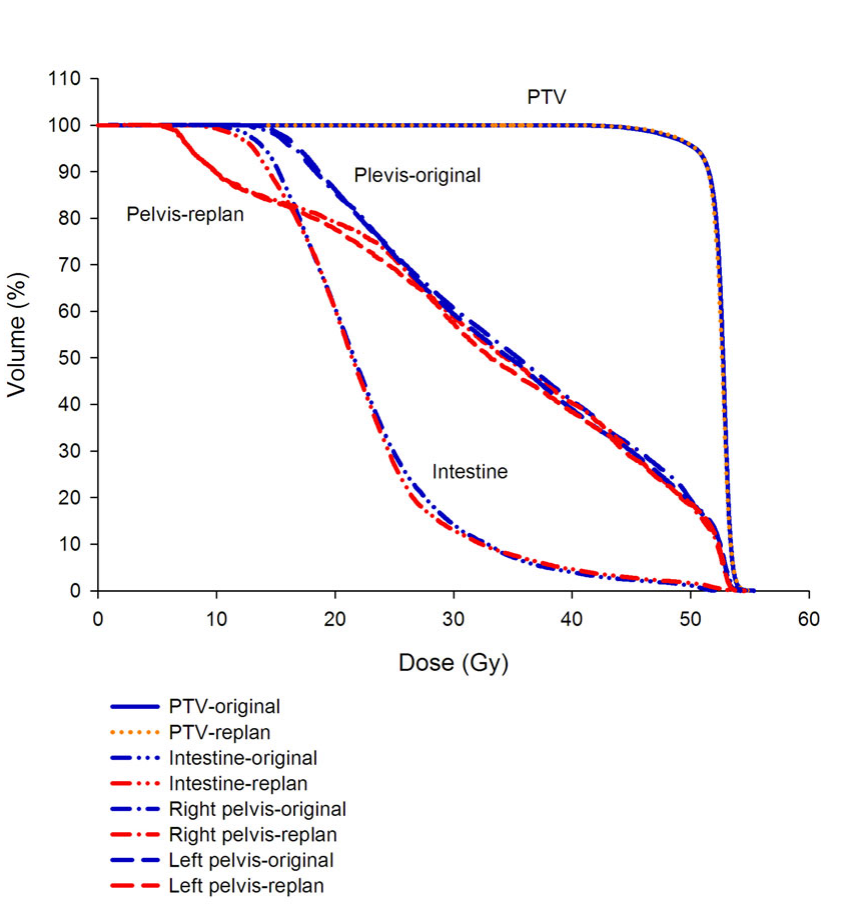
**Dose-volume histogram of pelvic bone marrow under the similar PTV and intestine dose for one patient with original whole pelvic helical tomotherapy and giving V10 < 90%, V20 <80% replanning whole pelvic helical tomotherapy for comparisons**.

HT can deliver dose to bone marrow exactly in total marrow irradiation and reduce the dose to OARs around 51%-74% when compared with total body irradiation [13]. It implies that HT can manage bone marrow precisely, either targeting or sparing. Brixey *et al*. [8] reported that acute hematological toxicity was reduced with pelvic IMRT compared with four-field box techniques in gynecologic cancer patients undergoing chemotherapy. Mell *et al*. [28] also provided evidence of an association between the volume of pelvic BM receiving low-dose radiation (V10, V20) and pointed out the potential of bone marrow sparing-IMRT could diminish the chronic effects of RT on BM suppression, improving chemotherapy tolerance. In our study, the pelvic bones sparing technique did not perform in the original WPHT plan and the value of V10 for pelvic bones almost achieving 100% was noted. In our retrospective data, 40% of acute moderate hematological toxicities happened in the CCRT and 10% of subacute thrombocytopenia was noted in the following days. It is noted that the highly conformal doses distribute to target and large off-target low dose existing simultaneously in the HT plan. If we target pelvic bone marrow according to Brixey *et al*. [8] and set pelvic bone marrow optimal constraints directly, HT can provide as much bone marrow sparing in the low dose as we desired. (Fig. 2) Since June first, the following cervical cancer patients in our center were performed pelvic bone sparing technique with WPHT. Up to day, three locally advanced cervical cancer patients completed the treatment by WPHT concurrent with chemotherapy and only grade 1 or 2 acute hematologic toxicities during CCRT are noted. The encouraging results hints that targeting pelvic bones and setting optimal constraint for pelvic bones can potentially decrease the acute and subacute clinical toxicities when use WPHT.

There are some limitations in our current study. First, the small case number and the retrospective study design make any statistical conclusions very tentative. Second, the follow-up time is short so the long-term results need to keep closely follow-up. Third, we do not perform pelvic bones sparing within this study perhaps this is the reason for acute hematologic toxicities dominant therefore enroll more patients by limiting bone marrow radiation dose with WPHT technique in the future to confirm our observation is warranted.

## Conclusions

To sum up, whole pelvic helical tomotherapy provides feasible clinical results in patients with locally advanced cervical carcinoma. Long-term follow-up and to enroll more locally advanced cervical carcinoma patients by limiting bone marrow radiation dose with WPHT technique is warranted.

## Competing interests

We have no personal or financial conflict of interest and have not entered into any agreement that could interfere with our access to the data on the research, or upon our ability to analyze the data independently, to prepare manuscripts, and to publish them.

## Authors' contributions

All authors read and approved the final manuscript. CHH and PWS carried out all CT evaluations, study design, target delineations and interpretation of the study. CHH drafted the manuscript. MCW, SMH and CAC took care of cervical cancer patients. HYL made the treatment planning and carried out all WPHT and WPRT comparisons and evaluations. THT and YJC participated in manuscript preparation and study design. LYW and YPH gave advice on the work and carried out statistical analysis.

## Supplementary Material

Additional file 1 **Acute and subacute toxicity for locally advanced cervical cancer patients received chemotherapy concurrent with whole pelvic helical tomotherapy followed by brachytherapy**. Click here for file

Additional file 2 The rate of cervical carcinoma treated with concurrent chemoradiation using helical tomotherapy at the Far Eastern Memorial Hospital (FEMH) compared with selected published series. Click here for file
