# Supplementary material for: Whole pelvic helical tomotherapy for locally advanced cervical cancer: technical implementation of IMRT with helical tomothearapy
Source: Radiat Oncol. 2009 Dec 10;4:62. doi: 10.1186/1748-717X-4-62 (PMC2799427; doi:10.1186/1748-717X-4-62)
Supplement: Additional file 1 — Acute and subacute toxicity for locally advanced cervical cancer patients received chemotherapy concurrent with whole pelvic helical tomotherapy followed by brachytherapy. [file 1748-717X-4-62-S1.DOC]

Additional file 1 - Acute and subacute toxicity for locally advanced cervical cancer patients received chemotherapy concurrent with whole pelvic helical tomotherapy followed by brachytherapy

| *Toxicity | No. of patient (%) | | | | | | |
| --- | --- | --- | --- | --- | --- | --- | --- |
|  | Acute toxicity | | | | | | |
|  | Nausea/  Vomitine | Diarrhea | Genitourinary effects | Body weight loss | Other hematologic  effects | Leukopenia | Thrombocytopenia |
| Gr.1 | 10 (100) | 7 (70) | 10 (100) | 10 (100) | 7 (70) | 3 (30) | 5 (50) |
| Gr.2 | 0 | 2 (20) | 0 | 0 | 3 (30) | 4 (40) | 4 (40) |
| Gr.3 | 0 | 1 (10) | 0 | 0 | 0 | 3 (30) | 1 (10) |
| Gr.4 | 0 | 0 | 0 |  | 0 | 0 | 0 |
| Gr.5 | 0 | 0 | 0 |  | 0 | 0 | 0 |
| *Toxicity | Subacute toxicity | | | | | | |
|  | Gastrointestinal (GI) effects | | Genitourinary effects | Body weight loss | Other hematologic  effects | Leukopenia | Thrombocytopenia |
|  | Upper GI | Lower GI |  |  |  |  |  |
| Gr.1 | 10 (100) | 10 (100) | 10 (100) | 10 (100) | 10 (100) | 8 (80) | 9 (90) |
| Gr.2 | 0 | 0 | 0 | 0 | 0 | 2 (20) | 0 |
| Gr.3 | 0 | 0 | 0 | 0 | 0 | 0 | 1 (10) |
| Gr.4 | 0 | 0 | 0 | - | 0 | 0 | 0 |
| Gr.5 | 0 | 0 | 0 | - | 0 | 0 | 0 |

*The grade of toxicity is according to the Common Terminology Criteria for Adverse Events v3.0 (CTCAE v3.0).
