# Supplementary material for: Whole pelvic helical tomotherapy for locally advanced cervical cancer: technical implementation of IMRT with helical tomothearapy
Source: Radiat Oncol. 2009 Dec 10;4:62. doi: 10.1186/1748-717X-4-62 (PMC2799427; doi:10.1186/1748-717X-4-62)
Supplement: Additional file 2 — The rate of cervical carcinoma treated with concurrent chemoradiation using helical tomotherapy at the Far Eastern Memorial Hospital (FEMH) compared with selected published series. [file 1748-717X-4-62-S2.DOC]

Additional file 2. The rate of cervical carcinoma treated with concurrent chemoradiation using helical tomotherapy at the Far Eastern Memorial Hospital (FEMH) compared with selected published series.

| Selected published series | Modality for whole pelvis | OARs | | | | | | | | | | |
| --- | --- | --- | --- | --- | --- | --- | --- | --- | --- | --- | --- | --- |
|  |  | Intestine | | | Bladder | | | Rectum | | | Pelvic bone | |
|  |  | V50.4 | V40 | V30 | V45 | V40 | V30 | V50.4 | V40 | V30 | V20 | V10 |
| Georg *et al.* [24] | IMRT | 4.6% | 39.4% | - | - | - | - | 12.3% | 27.0% | - | - | - |
| Menkarios *et al.* [25] | IMRT | - | 25.0% | 40.1% | - | 65.2% | 87.8% | - | - | - | - | - |
| Mell *et al.* [26] | IMRT | - | 39.7% | 57.4 | - | 73.6% | 91.5% | - | 83.7% | 86.7% | 57.5% | 76.5% |
| Roeske et al. [27] | IMRT | 0.5% | 26.0% | 45.3% | 76.6% | 86.1% | 96.5% | - | 73.9% | 94.5% | - | - |
| FEMH,  Taiwan | HT | 0.4% | 4.9% | 23.5% | 49.1% | 57.9% | 75.7% | 37.2% | 68.3% | 82.2% | 79.0% | 99.9% |

*Abbreviations:*

IMRT = Intensity-modulated radiotherapy; HT = helical tomotherapy; FEMH = Far Eastern Memorial Hospital. **Vx** = the **percent volume of organ with radiation dose more than x Gy.**
